# Synthesis of high-voltage cathode material using the Taylor-Couette flow-based co-precipitation method

**DOI:** 10.3389/fchem.2023.1195170

**Published:** 2023-04-24

**Authors:** Junghwan Lee, Young-Woong Song, HyoChan Lee, Min-Young Kim, Jinsub Lim

**Affiliations:** ^1^ Korea Institute of Industrial Technology (KITECH), Gwangju, Republic of Korea; ^2^ Department of Materials Science and Engineering, Chonnam National University, Gwangju, Republic of Korea

**Keywords:** spinel structure, co-precipitation, taylor flow, LNMO cathode material, LIB

## Abstract

LiNi_0.5_Mn_1.5_O_4_ (LNMO), a next-generation high-voltage battery material, is promising for high-energy-density and power-density lithium-ion secondary batteries. However, rapid capacity degradation occurs due to problems such as the elution of transition metals and the generation of structural distortion during cycling. Herein, a new LNMO material was synthesized using the Taylor-Couette flow-based co-precipitation method. The synthesized LNMO material consisted of secondary particles composed of primary particles with an octahedral structure and a high specific surface area. In addition, the LNMO cathode material showed less structural distortion and cation mixing as well as a high cyclability and rate performance compared with commercially available materials.

## 1 Introduction

**FIGURE 1:**
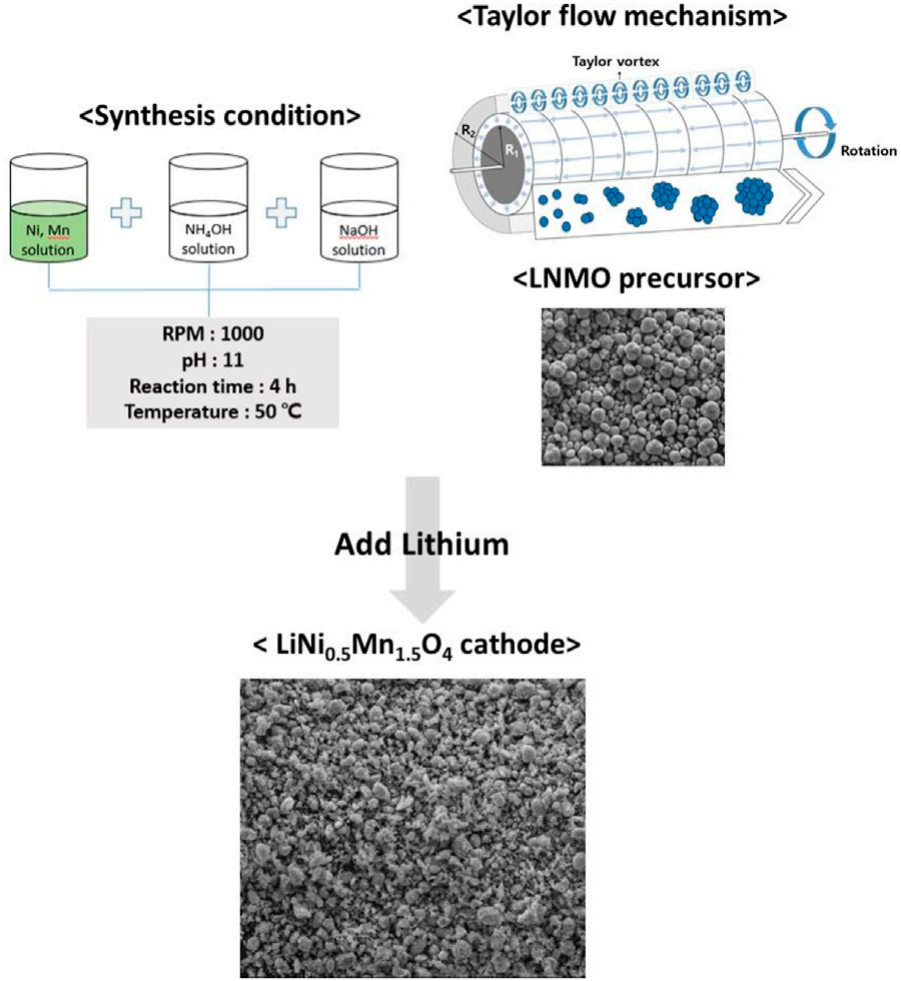
Schematic diagram of synthesis condition of co-precipitation and the Taylor-Couette flow mechanism.

Interest in the development of next-generation cathode materials with high energy and power density for lithium-ion secondary batteries applicable in large-scale energy storage and electric vehicle (EV) is increasing (Tarascon and Armand, 2001; Yang et al., 2013a; Yang et al., 2013b). A lithium-ion secondary battery consists of a cathode, anode, electrolyte, and a separator, and its capacity and power are determined by the cathode material (Li et al., 2017). One such material is LiNi_0.5_Mn_1.5_O_4_ (LNMO), a good alternative cathode material due to the fact that Li^+^ is reversibly intercalated at a potential of approximately 4.6–4.85 V (vs. Li/Li^+^) in the redox reaction of Ni^2+^/Ni^3+^ and Ni3+/Ni^4+^, increasing the energy density (650 Wh Kg^−1^) by around 20%–30% compared to that of conventional LiCoO_2_ and LiFePO_4_ materials (Scott et al., 2008; Liu et al., 2014). However, the presence of Mn^3+^ ions in LNMO results in the dissolution of Mn^2+^ ions (2Mn^3+^ → Mn^4+^ + Mn^2+^) during lithiation/delithiation at high operating voltage (>4.5 V) and temperature (>40°C), as well as in Jahn-Teller distortion; as a result, a significant capacity reduction occurs (Kunduraci and Amatucci, 2006; Li et al., 2007; Liu et al., 2010; Shin et al., 2012; Manthiram et al., 2014). In order to address these problems, various studies have investigated the development of a high-voltage electrolyte (Lee et al., 2019; Choi et al., 2015; Li et al., 2020a; Wang et al., 2016), surface coating, addition of a doping material (Teertstra et al., 2011; Wang et al., 2011; Sun et al., 2016; Wang et al., 2017; Bhuvaneswari et al., 2019), nano-sized particles (Lin et al., 2014; SONE et al., 2020), and low-temperature synthesis (<700°C) (Manthiram et al., 2014). Among these, surface coating and doping material addition have been shown to be relatively successful but, to effectively apply these methods, further research is essential to optimize the performance of basic LNMO. LNMO can be synthesized through the solid-state (Hagh and Amatucci, 2010; Kim et al., 2014a), co-precipitation (Feng et al., 2013; Li et al., 2016), sol-gel (Kunduraci et al., 2006; Kim et al., 2014b), and hydrothermal methods (Wang et al., 2015; Liu et al., 2016). Although the solid-state reaction is simple, it is difficult to control the size and shape of particles. Furthermore, while the sol-gel and hydrothermal processes can control the size and shape of particles, the sol-gel method is suboptimal for bulk production and the hydrothermal process is complicated, since a non-uniform crystal phase is formed. In contrast, co-precipitation is the primary method used for the synthesis of cathode material precursors since the particle formation process can be controlled, the particle size can be adjusted, and synthesis in ion units can create a homogeneous product (Cho et al., 2005; Vu and Lee, 2015). Various reaction systems have been used for the co-precipitation method. In general, a batch reactor and a continuous stirred tank reactor (CSTR) capable of uniformly controlling particle size are used as the reaction system. Batch reactors and CSTRs have the advantages of low cost, good mixing at the atomic level, and eco-friendliness. To highlight the importance of good mixing, research investigating the control of particle size distribution during co-precipitation through changes to the shape of the impeller is being conducted. However, controlling the strong energy flow generated near the impeller remains challenging (Jiang et al., 2017; Zhu et al., 2019). To overcome this challenge, we synthesized a precursor of LNMO cathode material via co-precipitation using a Taylor-Couette flow without an impeller. The Taylor-Couette flow has a unique flow characteristic in which an external or internal cylinder rotates when fluid flows between two cylinders in the direction of rotation. Furthermore, centrifugal and Coriolis forces, a virtual force acting on a rotating object similar to the centrifugal force, where the strength of the force is proportional to the speed of the moving body and acts perpendicularly to the direction of motion, affect the fluid of the internal cylinder; the fluid flows out in the direction of the external cylinder. Then, as the stirring speed increases, it becomes increasingly unstable, forming a vortex rotating in opposite directions along the axis, called a Taylor vortex (Figure 1) (Heo et al., 2021). Recently, many materials with dense and uniform distribution characteristics have been synthesized using a Taylor-Couette flow. However, to our knowledge there are no reports of synthesis of LNMO spinel using a Taylor-Couette flow. Therefore, in this study we synthesized a precursor of LNMO co-precipitated in a Taylor-Couette flow and compared it with commercial LNMO (C-LNMO) using various analytical methods, and its electrochemical properties were analyzed and compared.

## 2 Materials and methods

### 2.1 LiNi_0.5_Mn_1.5_O_4_ synthesis

**FIGURE 2:**
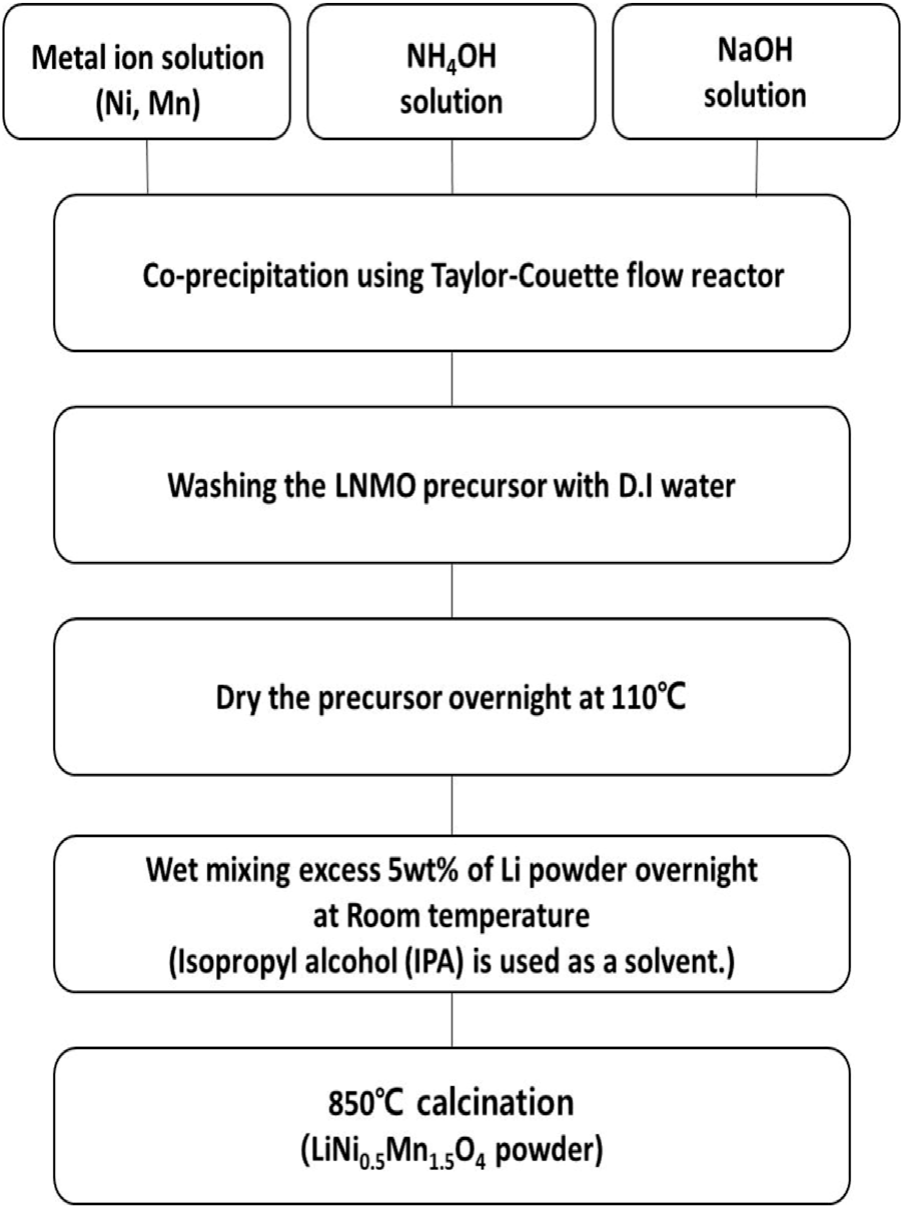
Flow chart of LiNi_0.5_Mn_1.5_O_4_ cathode material synthesis.

A spherical precursor of LNMO was synthesized through a continuous-type co-precipitation method using LCTR-tera 3,100 equipment manufactured by Laminar Co., Ltd. A metal aqueous solution consisting of 0.5 mol L^−1^ NiSO_4_.6H_2_O and 1.5 mol L^−1^ MnSO_4_.H_2_O were slowly pumped into a 1 L continuous-type reactor to which appropriate amounts of deionized water, NH_4_OH, and NaOH solutions were added without using an inert gas. Simultaneously, 4 mol L^−1^ of each a NaOH (aq.) and a NH_4_OH solution mixed with DI water at a ratio of 1:1 (aq.) were separately pumped into the reactor as chelating agents. A metal aqueous and a NH_4_OH aqueous solution were pumped into the reactor at a flow rate of 2 mL min^−1^ and 0.5 mL min^−1^, respectively. The NaOH aqueous solution pump was connected to a sensor that adjusted the flow rate based on the pH value. We set the pH value to 11 pH and maintained a residence time of 4 h. The rotation speed of the inner cylinder was set at 1,000 rpm. The precursor LNMO powder was obtained after washing, filtering, and drying samples at 110°C for 12 h. The dried powder was wet mixed with a Li source of more than 5% LiOH using isopropyl alcohol as a solution and then calcined at 850°C for 10 h in an O_2_ atmosphere. The synthesis process is shown in Figure 2. A commercial sample, C-LNMO, was prepared by Toshima MFG Co., Ltd. Using a solid-state method for comparative analysis.

### 2.2 Structural and physical characterization

The obtained samples were analyzed via X-ray diffraction (XRD) using an X’pert Pro X-ray diffractometer (PANalytical) with Ni-filtered Cu Kα radiation (*λ* = 1.5406 Å) operating at 40 kV and 30 mA within a scanning angle (2 θ) range of 10°–80° in 0.01° steps. Rietveld refinement obtained more detailed structural information for the unit cell using high-score plus software. The particle morphology characteristics of all samples were determined using field emission scanning electron microscopy (FE-SEM; S-4700 instrument, HITACHI). The surface areas of the samples were analyzed through the Brunauer–Emmett–Teller method using a Belosrp mini II (BEL) instrument under N_2_ gas. X-ray photoelectron spectroscopy (XPS) was performed at NEXSA (Thermo) using an Al Kα X-ray source.

### 2.3 Electrochemical measurements

The electrochemical performance of C-LNMO and TC-LNMO was examined using a half-cell with a Li anode. For electrochemical measurements, a stoichiometric 92:5:3 ratio of active material to conductive materials (Super P) to PvdF binder (KF7208) was used to fabricate the electrode, and the slurry was mixed using a Thinky Mixer (ARE-310). The stoichiometrically mixed slurry was cast onto an Al foil as a current collector and then dried at 110°C for 12 h in a conventional oven, thus forming the cathode. The mass loading of all the cells was controlled at approximately 5 ± 0.5 mg cm^−2^. A 2032 coin-type cell, consisting of the cathode and Li metal anode separated by a polymer membrane mixed with glass fiber, was fabricated in a dry room and aged for 10 h before electrochemical measurements were performed. The electrolyte employed was a 1:1:1 mixture of ethylene carbonate (EC), dimethyl carbonate (DMC), and diethyl carbonate (DEC) containing 1 M LiPF6 salt. Galvanostatic testing (WBCS 3,000, Wonatech, Korea) of the coin-type cell was performed over a potential range of 3.5–4.9 V vs. Li/Li^+^ at different charge/discharge current densities and 25°C. Electrochemical impedance spectroscopy (EIS) was performed at 25°C using a Bio-Logic Science instrument (SP-240) to determine the variation in the resistance of the assembled coin cells. The cells were tested at a pristine state and after cycles, with examination at each cycle in the 1 MHz−100 mHz frequency range.

## 3 Results and discussion

**FIGURE 3:**
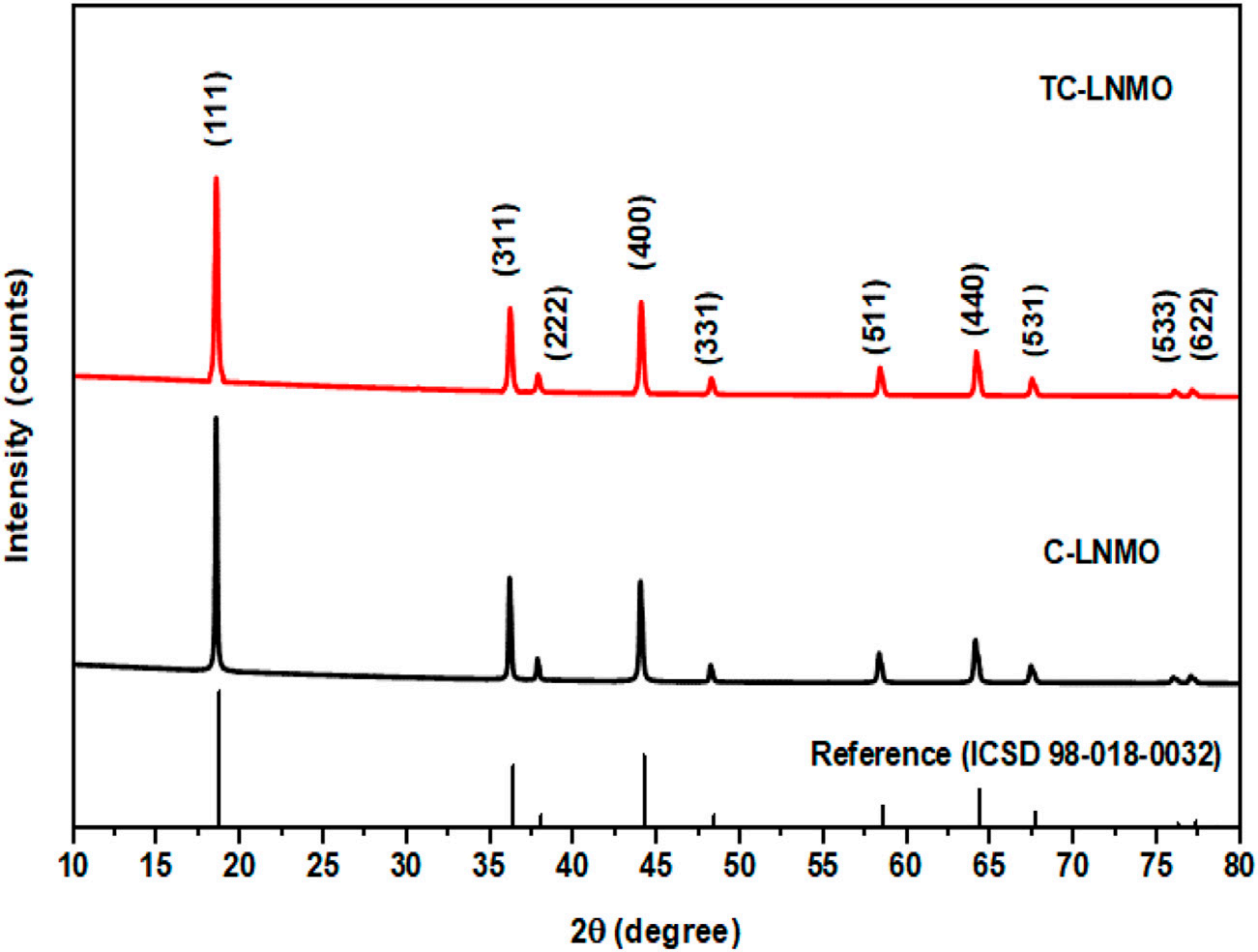
Powder XRD pattern of commercial LNMO and synthetic LNMO samples with reference peak of spinel structure.

The structural properties of C-LNMO synthesized using the solid-phase method and TC-LNMO synthesized using the co-precipitation method and Taylor–Couette flow were analyzed through XRD. The XRD patterns of the samples are shown in Figure 3 and conformed to the LiNi_0.5_Mn_1.5_O_4_ spinel structure with a disordered space group of Fd3-m (ICSD# 98-018-0032). Table Ⅰ summarizes the refined XRD results for C-LNMO and TC-LNMO. According to Table 1, the lattice parameters of C-LNMO and TC-LNMO were 8.1769 and 8.1766 Å, respectively, which was similar to the reference value of 8.171 Å. The degree of cation mixing in LiNi_0.5_Mn_1.5_O_4_ cathode materials can be obtained from the intensity ratio of I_(111)_/I_(311)_ (Liu et al., 2018). The I_(111)_/I_(311)_ values of C-LNMO and TC-LNMO were 2.454 and 2.479, respectively. The relatively high intensity ratio of TC-LNMO indicated that the degree of cation mixing was reduced and the occupancy of transition metals in tetrahedral sites was low (Wang et al., 2013). In addition, the structural distortion characteristics of LNMO can be inferred from the intensity ratio of I_(311)_/I_(400)_ (Li et al., 2018). The I_(311)_/I_(400)_ ratio of TC-LNMO was relatively lower than that of C-LNMO, indicating that TC-LNMO samples showed relatively less distortion. The BET specific surface areas of the C-LNMO and TC-LNMO samples were 0.58 and 2.61 m^2^ g^−1^, respectively. A high specific surface area can result in a high contact area between the electrode and electrolyte, resulting in rapid diffusion of Li^+^ ions (Wu et al., 2021). Therefore, these characteristics are expected to improve the electrochemical performance of TC-LNMO cathode material.

**TABLE 1:** Lattice parameters of LNMO samples obtained through Rietveld-refinement and surface area values obtained through BET.

Samples	Lattice parameter α(Å)	V (Å^3^)	I_(111)_/I_(311)_	I_(311)_/I_(400)_	Surface area (m^2^ g^−1^)
C-LNMO	8.1769	546.726	2.454	1.067	0.58
TC-LNNIO	8.1766	546.660	2.479	1.021	2.61

**FIGURE 4:**
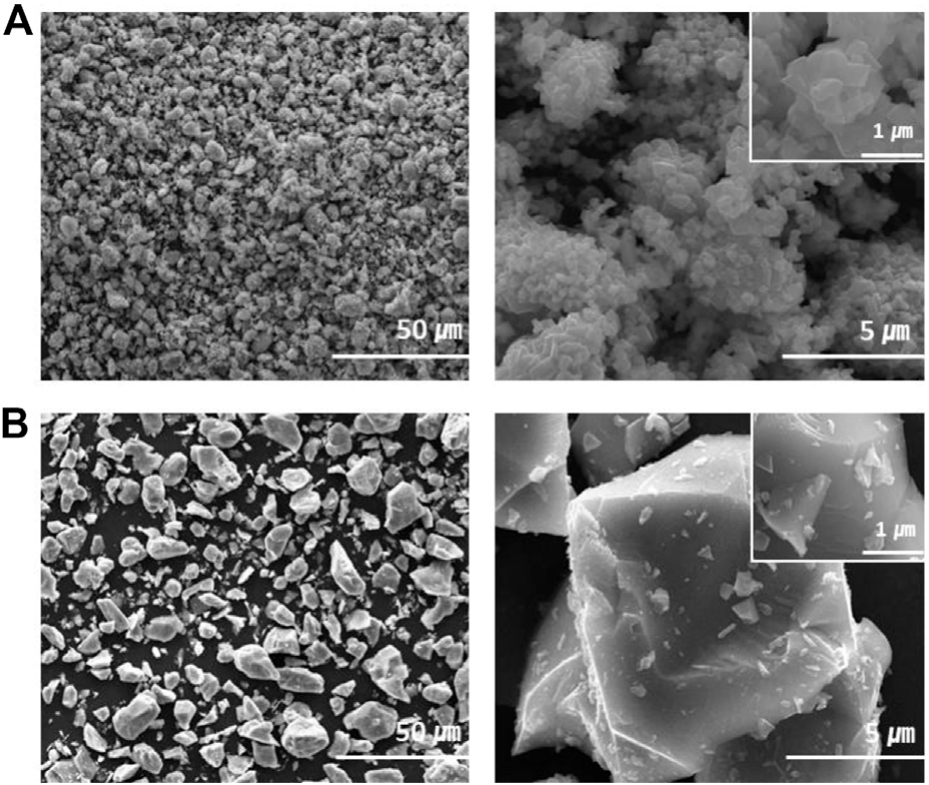
FE-SEM image of **(A)** synthestic LNMO and **(B)** commercial LNMO.

SEM images of the prepared morphologies of C-LNMO and TC-LNMO are shown in Figures 4A, B. The C-LNMO particles were composed of micro-sized particles, whereas the TC-LNMO sample was composed of secondary particles formed by the aggregation of nano-sized primary particles. In addition, it was confirmed that the TC-LNMO nanoscale primary particles formed an octahedron. It is suggested that a cathode with such a hierarchical surface is advantageous for electrolyte infusion and can improve the rate performance of the cathode active material (Zhu et al., 2014).

**FIGURE 5:**
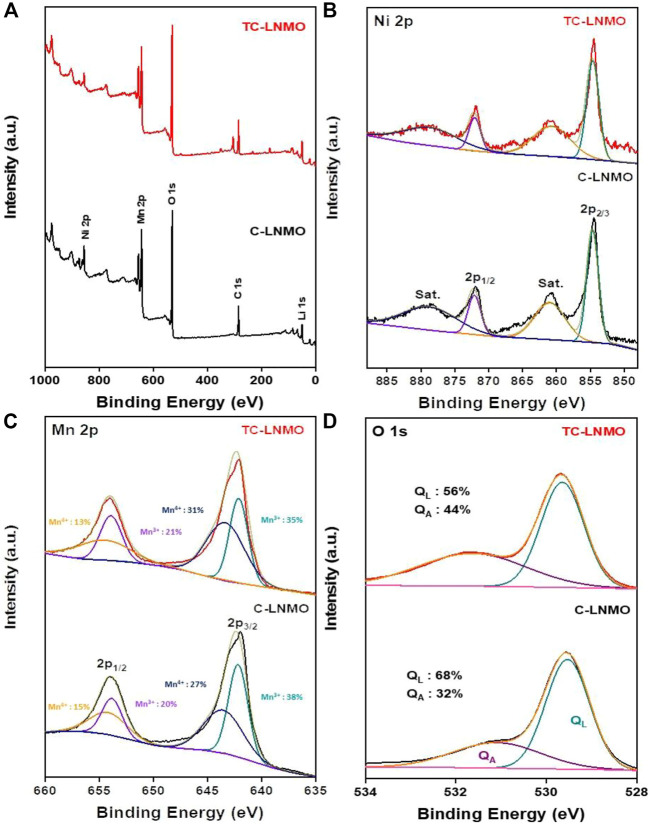
XPS spectra of C-LNMO and TC-LNMO samples. **(A)** overall spectrum, **(B)** Mn 2p, **(C)** Ni 2p and **(D)** O 1s regions.

Figure 5 summarizes the XPS results for the chemical composition of the particle surfaces of C-LNMO and TC-LNMO. In Figure 5A, it can be seen that the entire XPS spectrum appeared without impurities based on C 1s. In Figure 5B, both C-LNMO and TC-LNMO samples show Ni 2p spectra at 854.3 and 871.9 eV, respectively, due to the presence of Ni^2+^. Furthermore, the two satellite peaks that appeared at 861.0 and 878.8 eV could be attributed to the multiple splitting of the energy levels of nickel oxide (Ni-O) (Yang et al., 2014). As shown in Figure 5C, the peaks originating from Mn 2P appeared at 642.3 and 654.0 eV. The ratio of Mn^3+^ to Mn^4+^ in the fitted spectra of Mn 2P_3/2_ differed between C-LNMO and TC-LNMO. The relative ratio of Mn^3+^/Mn^4+^ was relatively low in TC-LNMO compared to that in C-LNMO. In general, the presence of excessive Mn^3+^ in LiNi_0.5_Mn_1.5_O_4_ causes Jahn-Teller distortion, which affects the elution of Mn ions and battery cycle characteristics (Liu et al., 2017). However, it has been reported that an appropriate Mn^3+^ content in the disordered Fd-3 m space group is beneficial for improving the performance of the material by increasing the Li^+^ ion diffusion rate and electronic conductivity (Duncan et al., 2014). Therefore, a relatively low Mn^3+^/Mn^4+^ ratio is useful for reducing Mn^2+^ formation while maintaining an adequate Mn^3+^ content (Han et al., 2022). Figure 5D shows the two main peaks (529.7 and 531.7 eV) of O 1s attributed to surface crystal lattice oxygen (Q_L_) and surface-absorbed oxygen (Q_A_). The broad peak at 529.7 eV is attributed to the crystal network, and the sharp peak at 531.7 eV is attributed to the adsorbed oxygen on the material surface. The Q_L_ and Q_A_ of C-LNMO and TC-LNMO were 68% and 32%, and 56% and 44%, respectively. This reduction in surface-absorbed oxygen is expected to enable high capacity and stable cycle characteristics (Li et al., 2019).

**FIGURE 6:**
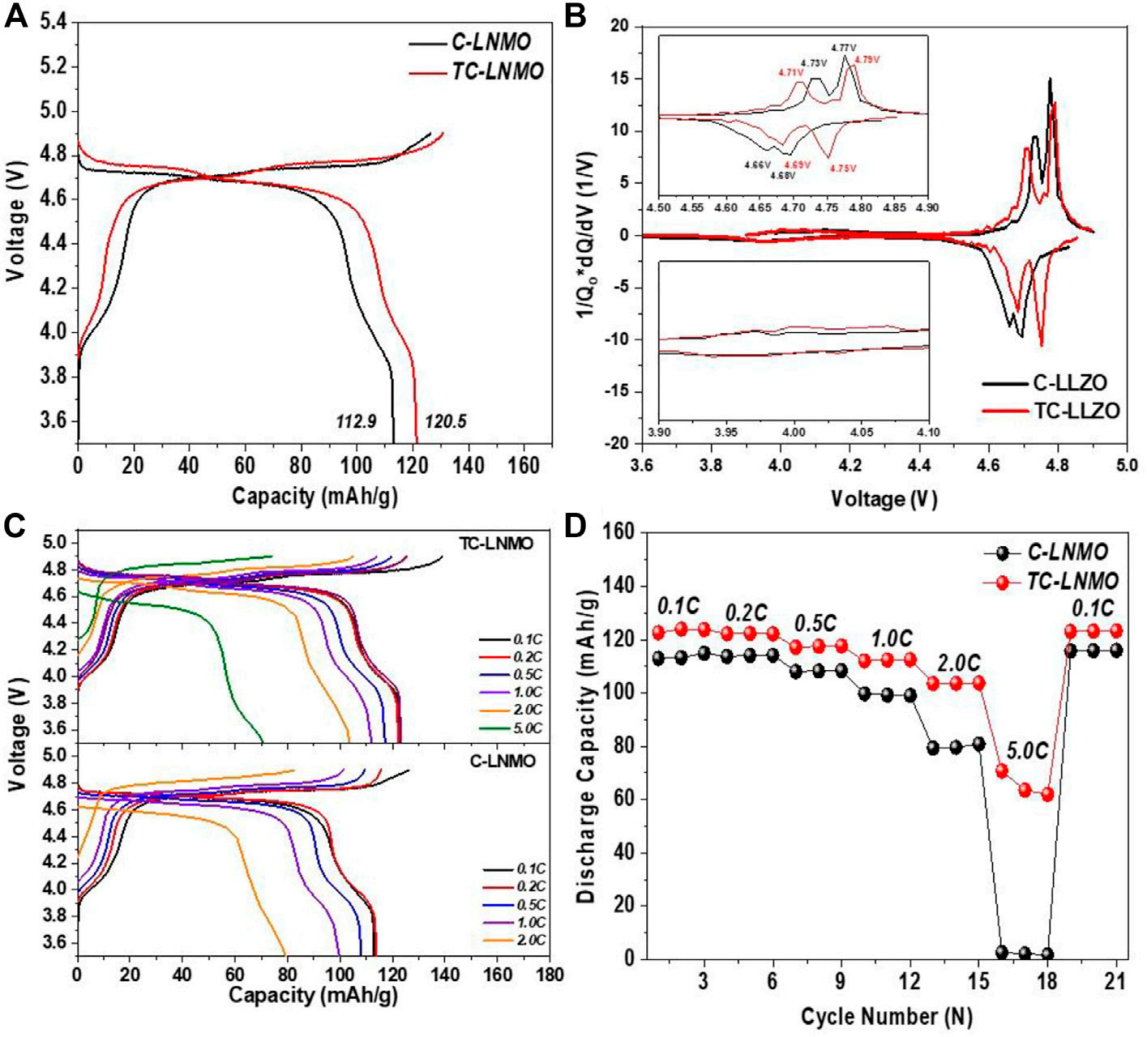
Electrochemical evaluation results of C-LNMO and T-cLNMO measured in the voltage range of 3.5–4.9 V at 0.1°C current density at 25°C: **(A)** Initial charge/discharge curves. **(B)** dQ/dV curves corresponding to the initial voltage profile. **(C)** Galvanostatic discharge curves at different C-rates. **(D)** Rate capability from 0.1°C to 5 C.

The charge/discharge tests of C-LNMO and TC-LNMO were evaluated in the 3.5–4.9 V cut-off range using a manufactured 2032 coin-type cell. Figure 6A shows the initial charge/discharge curves of C-LNMO and TC-LNMO. In both samples, a plateau was observed at 4.0 V due to Mn^3+^/Mn^4+^ redox coupling, and a difference between the Ni^2+^/Ni^3+^ and Ni^3+^/Ni^4+^ plateaus was clearly visible at 4.6 V, indicating a disordered Fd-3 m phase (Wu et al., 2017). The initial discharge capacities of C-LNMO and TC-LNMO were 112.9 and 120.5 mAh/g, respectively, highlighting that TC-LNMO had a relatively higher discharge capacity. Figure 6B shows the dQ/dV curves of the initial voltage profiles of C-LNMO and TC-LNMO. In the dQ/dV curves of C-LNMO and TC-LNMO, the difference in polarization due to Ni^2+^/Ni^3+^ and Ni^3+^/Ni^4+^ redox reactions in the range of 4.6–4.8 V is shown. TC-LNMO had a redox coupling difference of 0.2 and 0.4 V between Ni^2+^/Ni^3+^ and Ni^3+^/Ni^4+^, respectively, but C-LNMO had one of 0.6 and 0.9 V, enabling TC-LNMO to have a lower polarization. Figures 6C, D shows the results of the rate capability analysis of C-LNMO and TC-LNMO samples recovered to 0.1 C after three cycles for each C-rate from 0.1 to 5 C. At all C-rates, TC-LNMO exhibited a high discharge capacity, and the 2 C capacity retention rate compared to the 0.1 C rate of C-LNMO and TC-LNMO was 70.2% and 84.6%, respectively. In addition, at 5 C, the C-LNMO sample exhibited rapid capacity fading, whereas the TC-LNMO exhibited a discharge capacity of 70.8 mAh/g. These results indicate that the use of TC-LNMO is advantageous for the fabrication of high-power batteries.

**FIGURE 7:**
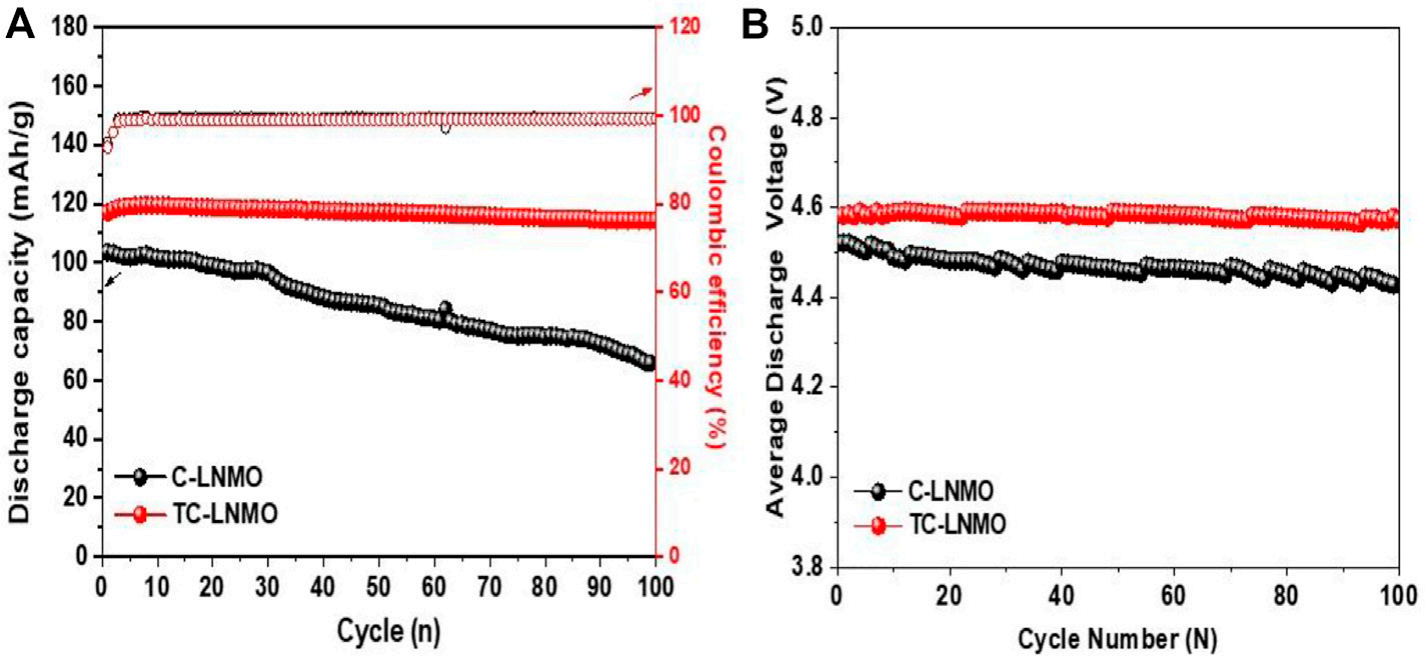
Cycling performances of C-LNMO and TC-LNMO samples. **(A)** Capacity and Coulombic efficiency for 100 cycles at 1°C rate. **(B)** The average discharge voltage curves.

Figure 7A shows the results of the evaluation of the cyclability characteristics of the C-LNMO and TC-LNMO samples at 1 C. The 100 cycle capacity retention rates of the C-LNMO and TC-LNMO samples were 63.8% and 97.7%, respectively, indicating that TC-LNMO had high-capacity retention characteristics. Figure 7B shows the average discharge voltage of the C-LNMO and TC-LNMO samples during 100 cycles. The high electrochemical properties of TC-LNMO are attributed to the higher specific surface area compared to C-LNMO, the hierarchical morphological characteristics, and the proper mixing of Mn^3+^ and Mn^4+^ shown in XPS. The average discharge voltage is an important factor in measuring the energy density of a battery. The initial discharge voltages of C-LNMO and TC-LNMO were 4.52 and 4.58 V and the 100 cycle discharge voltages were 4.42 and 4.57 V, respectively. TC-LNMO showed a voltage reduction of only 0.01 V after 100 cycles, suggesting that batteries using TC-LNMO can maintain high energy density, even after long-term cycling.

**FIGURE 8:**
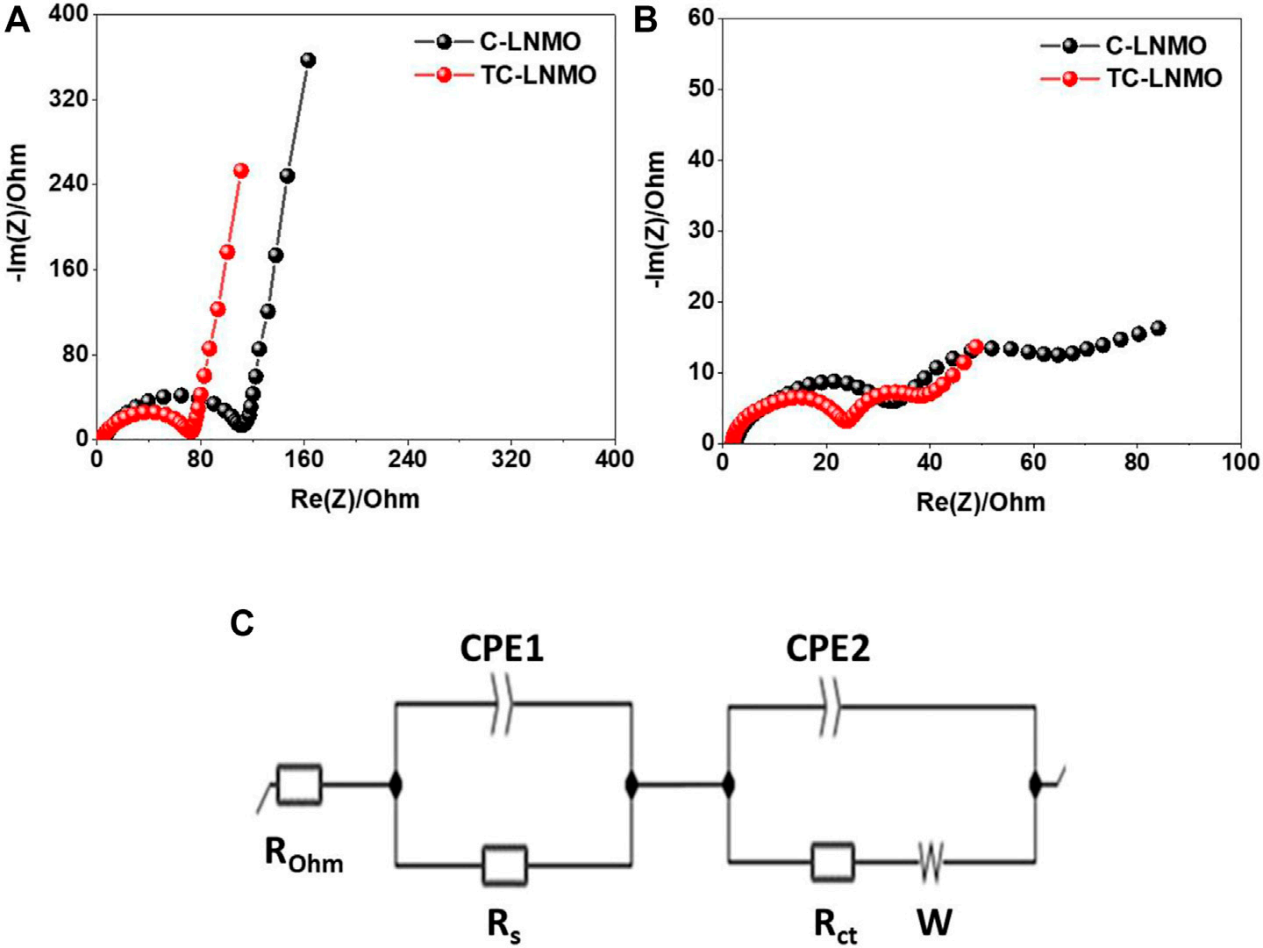
EIS spectra of the assembled coin cell recorded over the 100 mHz−1 MHz range **(A)** before cycling **(B)** after cycling **(C)** an equivalent circuit model.

EIS was performed to analyze the resistance of the internal elements of the cells prepared using the C-LNMO and TC-LNMO samples. The EIS spectra of C-LNMO and TC-LNMO measured at each cycle are summarized in Figures 8A, B, respectively. The equivalent circuit corresponding to the EIS spectra is shown in Figure 8C, where R_ohm_ is the ohm resistance corresponding to the intercept of the semicircle in the high-frequency range, and R_s_ is the resistance of the surface, which corresponds to the semicircle in the high-frequency range. R_ct_ is the charge transfer resistance corresponding to the semicircle of the medium-frequency region, and the inclined line in the low-frequency region represents the Warburg impedance (W) (Li et al., 2020b; Wu et al., 2021). The resistance values of the C-LNMO and TC-LNMO samples are listed in Table 2. Since the EIS spectrum of the pristine cell was sampled before the film was formed between the electrolyte and the electrode, the difference between the R_s_ and R_ct_ resistances was too small; therefore, its entirety was used for comparison. In contrast, the R_s_ and R_ct_ resistances were clearly observed after cycling. This differences in resistance can be related to the cation mixing in TC-LNMO shown in the XRD analysis (Figure 3) and the appropriate Mn^3+^ content shown in the XPS analysis (Figure 5). Another effect may be due to the difference in the reaction area according to the specific surface area of the particles shown in the FE-SEM images (Figure 4). Therefore, the low internal resistance of TC-LNMO proves that it is a cathode material with excellent rate and cycling characteristics.

**TABLE 2:** Resistance parameters of LNMO samples before and after charging and discharging.

Before cycle			After cycle		
Sample	R_ohm_	R_s_ + R_et_	R_ohm_	R_s_	R_ct_
C-LNMO	3.614	112.407	2.442	33.305	67.515
TC-LNMO	2.463	72.301	1.845	24.036	39.876

## 4 Conclusion

We synthesized LNMO cathode active materials using the Taylor-Couette flow-based co-precipitation method. The XRD analysis confirmed that the synthesized LNMO sample had a disordered Fd-3 m space group. In addition, cation mixing and structural distortion occurred less than in commercially available LNMO and showed a high specific surface area. FE-SEM images confirmed that TC-LNMO was composed of octahedron-shaped primary particles and formed spherical particles. The TC-LNMO sample had a capacity of 120.5 mAh/g at 0.1 C and a higher capacity than commercial products at 5 C. In addition, it had a high-capacity retention rate of 97.7% and retained an average discharge voltage, even after 100 cycles, which is believed to result from it containing a sufficient Mn^3+^ content. Therefore, this study outlines a novel approach for the synthesis of next-generation, high-voltage battery materials.

## Data Availability

The original contributions presented in the study are included in the article/supplementary material, further inquiries can be directed to the corresponding author.
